# Human DNA extraction from whole saliva that was fresh or stored for 3, 6 or 12 months using five different protocols

**DOI:** 10.1590/1678-77572016-0046

**Published:** 2017

**Authors:** Thais Francini GARBIERI, Daniel Thomas BROZOSKI, Thiago José DIONÍSIO, Carlos Ferreira SANTOS, Lucimara Teixeira das NEVES

**Affiliations:** 1 Universidade de São Paulo, Hospital de Reabilitação de Anomalias Craniofaciais, Bauru, SP, Brasil.; 2 Universidade de São Paulo, Faculdade de Odontologia de Bauru, Bauru, SP, Brasil.

**Keywords:** Saliva, DNA, Spectrophotometry, Electrophoresis, Polymerase chain reaction

## Abstract

Saliva when compared to blood collection has the following advantages: it requires no specialized personnel for collection, allows for remote collection by the patient, is painless, well accepted by participants, has decreased risks of disease transmission, does not clot, can be frozen before DNA extraction and possibly has a longer storage time.

**Objective:**

and

**Material and Methods:**

This study aimed to compare the quantity and quality of human DNA extracted from saliva that was fresh or frozen for three, six and twelve months using five different DNA extraction protocols: protocol 1 – Oragene™ commercial kit, protocol 2 – QIAamp DNA mini kit, protocol 3 – DNA extraction using ammonium acetate, protocol 4 – Instagene™ Matrix and protocol 5 – Instagene™ Matrix diluted 1:1 using proteinase K and 1% SDS. Briefly, DNA was analyzed using spectrophotometry, electrophoresis and PCR.

**Results:**

Results indicated that time spent in storage typically decreased the DNA quantity with the exception of protocol 1. The purity of DNA was generally not affected by storage times for the commercial based protocols, while the purity of the DNA samples extracted by the noncommercial protocols typically decreased when the saliva was stored longer. Only protocol 1 consistently extracted unfragmented DNA samples. In general, DNA samples extracted through protocols 1, 2, 3 and 4, regardless of storage time, were amplified by human specific primers whereas protocol 5 produced almost no samples that were able to be amplified by human specific primers. Depending on the protocol used, it was possible to extract DNA in high quantities and of good quality using whole saliva, and furthermore, for the purposes of DNA extraction, saliva can be reliably stored for relatively long time periods.

**Conclusions:**

In summary, a complicated picture emerges when taking into account the extracted DNA’s quantity, purity and quality; depending on a given researchers needs, one protocol’s particular strengths and costs might be the deciding factor for its employment.

## Introduction

For large scale genetic studies, the amount and quality of DNA available from a sample is an essential requirement. Usually the preferred source for the collection of genetic material for these studies is peripheral blood^1^ because it yields large amounts of DNA (~10 to 15 µg *per* mL of blood)^16^ typically free of foreign DNA, yet this procedure is not optimum since venipuncture can be a painful experience and has the possibility of transmitting diseases^4^, requires trained personnel for collection, is commonly feared by participants causing volunteers to refuse to participate in research^14^ and the DNA in extracted blood degrades quickly without refrigeration and must typically be processed approximately seven days after storage^16^. Another important factor to consider when using blood samples is the presence of ferrous ions (Fe^2+^) that compete with Mg^2+^ ions, which can inhibit the polymerase chain reaction (PCR) techniques widely employed by molecular studies^1^. Additionally, there may be cultural barriers to extracting blood^11^, and exceptional care most be taken when sending blood samples from different locations. Lastly, analyzing genomic DNA from bone marrow transplant recipients is unfeasible.

In general, molecular analysis requires several processing steps^2^ with DNA extraction being one of the most important steps for the success of a molecular genetic study^13^. All the reasons stated above have led to searches for alternative methods to obtain genetic material for studies requiring DNA, with saliva being considered one of the best candidates^13^.

Briefly, the sublingual, parotid and the submandibular glands secrete saliva. Furthermore, the shedding of the superficial layer of epithelial cells in human oral mucosa that occurs approximately every 2.7 hours^5^ ultimately leads to saliva composed of ~75% epithelial cells (~430,000 cells *per* mL^5^) and ~25% leukocytes (2 to 136,000 cells *per* mL)^7^ ^,^ ^19^ depending on the oral health of the individual. Endler, et al.^7^ (1999) found on average, at least 58% of the epithelial cells present in collected saliva samples to be viable with intact genomic DNA^7^. However, their extraction protocol, based on Sambrook, et al.^17^ (1988), typically extracted the majority of DNA from leukocytes and they hypothesized that their protocol more easily extracted DNA from leukocytes compared to epithelial cells in their saliva samples from bone marrow transplant patients^17^. However, it remains to be studied if modern DNA extraction protocols differ in this respect from the protocol based on Sambrook, et al.^17^ (1988).

Dawes (2003), moreover, found that saliva from his volunteers typically contained ~430,000 epithelial cells *per* mL, and that, on average, each epithelial cell had approximately 80 to 100 bacteria attached^5^. Thus 1 mL of human saliva contains a mixture of DNA from approximately ~4.3x10^5^ epithelial cells, ~1.36x10^5^ leukocytes and ~1.7x10^7^ bacteria along with DNA from other microorganisms found in the oral cavity. Besides saliva, oral cells can be collected using a variety of methods, such as the following: swabs, cotton spit wads^6^, cytological brushes, mouthwash with saline and treated Guthrie cards^9^.

Other components of saliva such as enzymes, hormones, immunoglobulins and other biomolecules can also interfere with the quality and quantity of the genomic DNA extracted^14^. Overall, care should be used for both DNA extraction and the preservation of the saliva^14^. In particular, care should be exercised when examining the DNA from bone marrow transplant recipients since saliva samples commonly produce a chimeric mixture of donor and recipient DNA. Endler, et al.^7^ (1999) found chimeric DNA samples from 6 out of 8 saliva samples from bone marrow transplant recipients whereas Thiede, et al.^19^ (2000) found only donor DNA in approximately 10 to 15% of recipients from saliva samples^7^ ^,^ ^19^.

Ordinarily, however, saliva can be a good source of human DNA when compared to other alternative DNA sources. Saliva can be easily collected by untrained individuals and extracted DNA with a high molecular weight can be stored for long periods of time - up to 5 years at room temperature according to DNA Genotek (DNA Genotek; Ottawa, Ontario, Canada)^1^ ^,^ ^16^ ^,^ ^20^. Also, saliva collection is painless, with minimal risk of disease transmission. Since saliva collection is noninvasive, repeated collections are well tolerated by most patients^15^, and patients can send saliva samples by mail, thus, facilitating collection.

Küchler, et al.^12^ (2011) evaluated the yield and quality of genomic DNA obtained from fresh saliva and saliva stored for 4 and 8 days at room temperature using one protocol^12^. Their results indicated no significant difference between the different storage periods. However, there are a few studies that investigated the possible influence of freezing for long term storage^6^, which is of fundamental importance for the creation of a saliva bank in large research centers.

Currently, various DNA extraction kits are commercially available, which standardize efficient and convenient methods for obtaining genomic DNA from saliva^14^. Different extraction methods can yield different amounts of DNA with varying levels of purity^20^. Often both the quantity and quality of DNA directly influence the success and results of studies. Knowing the maximum and ideal storage durations for saliva samples is valuable for minimizing the loss of quantity and/or quality of DNA. Moreover, inferior DNA extraction can prevent the successful completion of an experimental study, therefore wasting time and money.

Thus, this study aims to evaluate the quantity and quality of genomic DNA obtained from cells present in fresh saliva and saliva frozen for three, six and twelve months using five different DNA extraction protocols.

## Material and methods

### Sample collection

Twenty people were invited to participate in this study. Eligibility criteria included healthy male or female adults aged ≥18 years. After explaining the purpose of the study and how to participate, a consent form was completed and signed by all participants. This study was approved by the Ethics Committee for Research from the Bauru School of Dentistry, University of São Paulo, Bauru, SP, Brazil, under protocol number 192/2011.

Briefly, participants expectorated at least 20 mL of unstimulated saliva into a sterile, 50 mL polyethylene tube at least 30 minutes prior to eating, drinking, smoking or kissing to minimize contaminates^2^. These collection tubes were maintained on ice and the saliva was aliquoted into sterile microcentrifuge tubes (1.5 mL). Next, each participant’s aliquoted samples were stored at -20°C for three (T3), six (T6) and twelve months (T12). The storage periods stipulated above for the whole saliva aimed to assess whether this freezing would affect the quantity and quality of genomic DNA obtained when compared to fresh saliva (T0).

### DNA extraction

The five genomic DNA extraction protocols used are described below. For all commercial kits, the manufacturers’ specific instructions were followed.

**Protocol 1** - Protocol using manual purification of DNA via the commercial kit Oragene™ (DNA Genotek OG-500; Ottawa, Ontario, Canada). This kit provides a collection tube with 1 mL of suspension buffer containing proprietary reagents that stabilize the whole saliva sample prior to DNA extraction. Specifically, approximately 1 mL of saliva was mixed with the collection buffer and 500 µL of this mixture was used for the DNA extraction. The final elution volume was 100 µL.

**Protocol 2** – Protocol using the QIAamp^®^ DNA Mini Kit (Qiagen^®^ 51306; Hilden, North Rhine-Westphalia, Germany). The DNA extraction protocol uses silica columns and no suspension buffer. Protocol 2 used 200 µL of saliva for the DNA extraction and had a final elution volume of 50 µL.

**Protocol 3** – Protocol for DNA extraction from whole saliva using ammonium acetate, adapted from Aidar and Line^1^ (2007). One modification to this protocol included collecting saliva without a suspension buffer^1^. Whole saliva was centrifuged in a 1.5 mL microcentrifuge tube at 10,000 *g* for 5 minutes. The supernatant was discarded and the pellet was resuspended in 1 mL of extraction buffer [10 mM Tris – HCl; pH 7.8; 5 mM EDTA; 0.55% sodium dodecyl sulfate (SDS)]. Next, 5 µL of proteinase K (20 mg/mL; Qiagen^®^ 19133; Hilden, North Rhine-Westphalia, Germany) were added to degrade proteins. The tubes were vortexed and incubated in a water bath at 56°C overnight. Then samples were centrifuged quickly (to collect all the liquid to the bottom), and 500 µL of 10 M ammonium acetate solution was added to the tubes, which were mixed manually for 3 to 5 minutes followed by centrifuging at 21,000 *g* for 15 minutes at room temperature. Then 500 µL of this supernatant was transferred to a new tube, and 540 µL of cold isopropyl alcohol was added followed by 15 seconds of vortexing. The samples were placed in a refrigerator for 2 hours and then centrifuged at 10,000 *g* for 20 minutes at room temperature. The supernatant was discarded with care to not re-suspend the pellet of DNA; 1 mL of cold 70% ethanol was added to the tubes, which were then centrifuged at 10,000 *g* for 5 minutes. The supernatant was again discarded, and the tubes were left open for 4 to 5 hours to evaporate the excess alcohol and then the DNA was hydrated in 50 µL of autoclaved deionized water (50 µL final elution volume).

**Protocol 4** – Protocol of DNA extraction from whole saliva with InstaGene™ Matrix (Bio-rad 7326030; Hercules, California, United States). Tubes containing 1.5 mL of whole saliva samples without any suspension buffer were vortexed for uniformity of the content and centrifuged at 10,000 *g* for 5 minutes at 4°C, the supernatant was discarded; 1 mL of physiological saline was added to the tubes which were then vortexed until the pellet was dissolved and then vortexed for an additional 30 seconds. The tubes were centrifuged at 10,000 *g* for 5 minutes at 4°C, and the supernatant was discarded. The same procedures with the addition of 1 mL of physiological saline were repeated twice more. Next, 200 µL of InstaGene Matrix was added, this mixture was vortexed for 30 seconds and the samples were incubated at 56°C for 30 minutes. The tubes were vortexed again for 10 seconds, and boiled at 100°C for 10 minutes and again vortexed for 10 seconds and centrifuged at 15,000 *g* for 5 minutes at 4°C. Finally, the supernatant with the extracted DNA was pipetted into a new tube leaving a final elution volume of approximately 170 µL.

**Protocol 5** – Protocol of DNA extraction from whole saliva with InstaGene™ Matrix (InstaGene™ Matrix diluted 1:1) using Proteinase K and 1% SDS. Tubes containing 1.5 mL of total saliva without any suspension buffer were vortexed for uniformity and centrifuged at 10,000 *g* for 5 minutes at 4°C, the supernatant was discarded, and 1 mL of physiological saline was added. The tubes were vortexed until the precipitate completely dissolved and then vortexed for an additional 30 seconds. The tubes were centrifuged at 10,000 *g* for 5 minutes at 4°C, and the supernatant was discarded. The same procedures with the addition of 1 mL of physiological saline were repeated twice more. Next, 100 µL of DNAse and RNAse free water and 100 µL of InstaGene™ (1:1) was added to the tubes, which were then vortexed for 30 seconds. Samples were incubated at 56°C for 30 minutes and vortexed for 10 seconds. The tubes were boiled at 100°C for 10 minutes and again vortexed for 10 seconds. Following this procedure, 2 µL of Proteinase K (40 µg) and 2 µL of 1% SDS were added to the samples and then vortexed and incubated at 65ºC for 30 minutes (every 10 minutes the tubes were mixed). Then, the tubes were vortexed again and centrifuged at 15,000 *g* for 5 minutes at 4°C. Finally, the supernatant containing the extracted DNA was pipetted into a new tube leaving a final elution volume of approximately 170 µL.

### Spectrophotometric analysis

A spectrophotometer at wavelengths of 260 nm and 280 nm (NanoDrop™ 1000, Thermo Fisher Scientfic; Waltham, Massachusetts, United States) was used to quantify and analyze the condition of each extracted DNA sample from saliva. This equipment provides two important measures: (1) concentration (ng/µL) and (2) purity (via the relative absorbance ratio of 260 nm/280 nm) for DNA with regards to proteins and RNA. Samples with a ratio closer to 1.8 indicate a relatively pure DNA sample. The 260/280 nm ratios appreciably lower than 1.6 are indicative of higher protein contaminates. Samples were considered pure if the absorbance ratio was between 1.6 and 2.0. This analysis requires 2 µL of each sample.

### Electrophoretic analysis

To further determine the quality and condition of the extracted DNA, samples of DNA from fresh saliva or saliva stored for 3, 6 and 12 months using five different extraction protocols for each time point were electrophoresed using a 0.8% agarose gel (14 mm x 11.6 mm x ~5 mm) in TAE buffer, Tris-acetate (200 mM) with EDTA (50 mM). After the DNA was extracted, it was stored for an additional 3 years before it was analyzed using electrophoresis. In particular, a standard molecular weight of 100 bp DNA ladder (Invitrogen; Waltham, Massachusetts, United States), a positive control (115 ng of DNA, 8 µL), a negative control (8 µL of ddH_2_O) and 8 µL samples from individuals were loaded onto each gel with 2 µL of loading buffer (NEOBIO products for laboratories, catalog number: NB-NT-40501; Botucatu, SP, Brazil). The samples were loaded in to two rows of wells (2 mm x 1 mm x ~4 mm) with 5 cm lanes and driven by a power supply (Loccus Biotechnologia LPS-300V; Cotia, SP, Brazil) for ~150 minutes using 40 mA, ~10 V *per* cm, at room temperature. After electrophoresis, the DNA on the gels was visualized using UV light (Sigma-Aldrich T2202; St. Louis, Missouri, United States), photographed using a digital camera (Canon Inc. PC1089; Ōta, Tokyo, Japan) and captured with Doc-ItLS (version 6.0.0) software (UVP; Upland, California, United States).

### PCR

**Figure 1:**
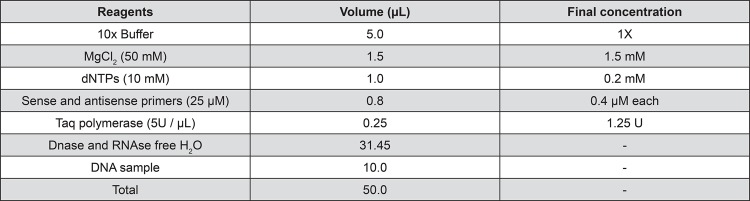
Reagents used to prepare a polymerase chain reaction (PCR) mixture

Conventional PCR was also used to investigate the following two additional aspects of the extracted DNA: (1) if any of the protocols introduced any variables that would inhibit DNA amplification and (2) using primers with 100% specificity for humans to verify if human DNA was extracted. Briefly, PCR was used to amplify exon 3 of the interferon regulatory factor 6 (*IRF6*) gene. The sequences of primers used were 5’-AGCTCTAGTAGATGGGAAAGGTG-3’ (sense strained) and 5’-CCAGAAAGGTCTGATGGTAGAAG-3’ (antisense strained) resulting in an amplified fragment of 302 bp. All the reagents used (Invitrogen PCR kit, catalog number 11615-010; Waltham, Massachusetts, United States) are outlined in Figure 1.

MgCl_2_: magnesium chloride

dNTPs: deoxynucleotides triphosphates

Conventional PCR was performed using a thermocycler (Thermo Electron Corporation PxE0.5; Waltham, Massachusetts, United States) with an initial denaturing step at 95°C for 4 min, followed by 40 cycles of amplification. Each amplification cycle consisted of denaturation for 30 seconds at 95°C, annealing for 30 seconds at 63°C and an extension for 1 minute at 72°C. The samples were incubated for an additional 7 minutes at 72°C and maintained at 4ºC until the time of removal.

To confirm amplification of the fragment, 8 µL of the PCR product from each sample was mixed with 2 µL of loading buffer (NEOBIO products for laboratories, catalog number: NBNT40501; Botucatu, SP, Brazil) and electrophoresed in a 2% agarose gel using the same procedures outlined above.

### Statistical analysis

Data were analyzed using Microsoft^®^ Excel 2002 (version 10.6871.6870), IBM^®^ SPSS^®^ statistics (version 20.0.0) and GraphPad Prism 5.0. Briefly, data were tested for normality using the Shapiro-Wilk test. Non-normally distributed data were compared using the Kruskal-Wallis test. Mann-Whitney U tests were employed to identify the specific differences between groups. Binary data (*e.g.* whether DNA tested within purity limits) were compared using the Pearson’s chi-squared test. Statistical significance was set at 0.05. Non-normally distributed data are represented by box-and-whisker plots reporting medians with interquartile ranges [IQRs].

## Results

**Figure 2:**
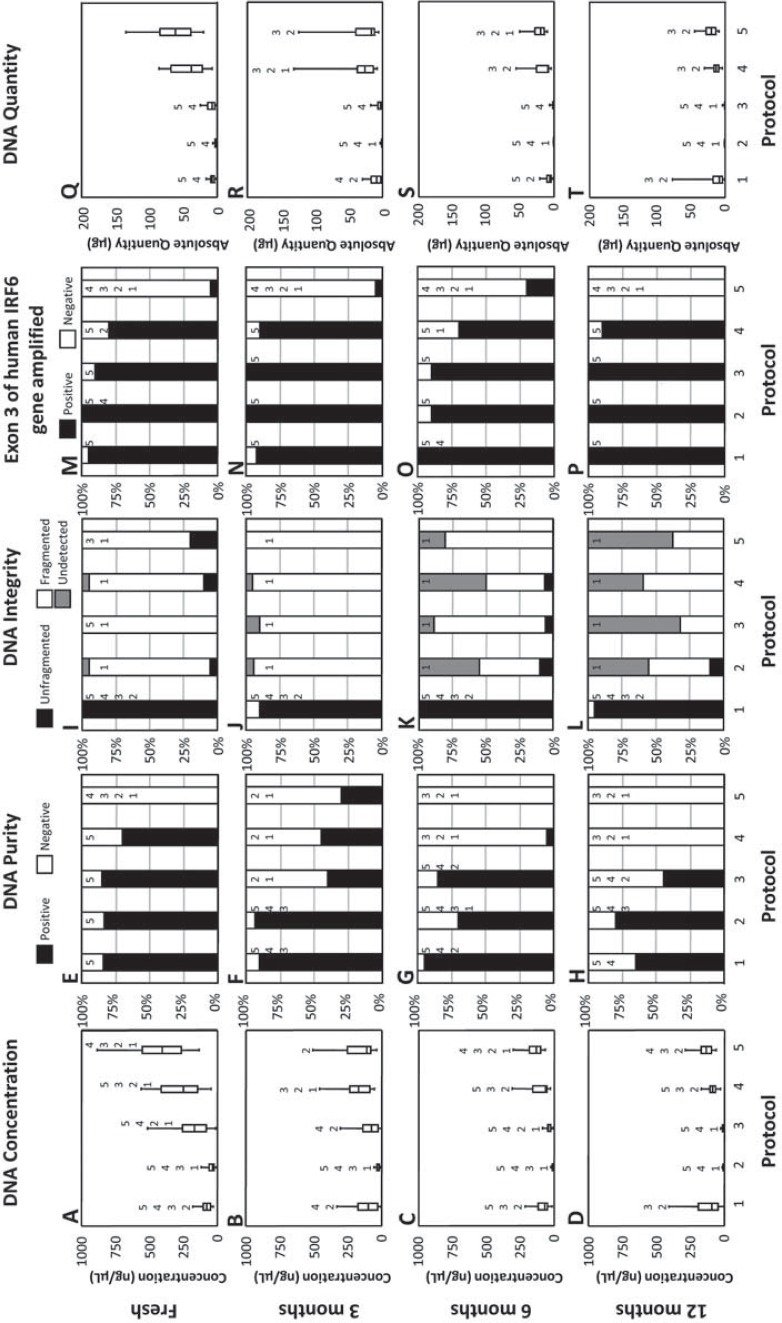
DNA quantity, purity, integrity and whether each sample contained amplifiable human DNA obtained from fresh saliva and saliva frozen at different time periods using five different extraction protocols. Protocol 1 (1) used the Oragene™ kit; protocol 2 (2) used the QIAamp® DNA Mini kit; protocol 3 (3) used ammonium acetate, protocol 4 (4) used the InstaGene™ Matrix kit; protocol 5 (5) used the InstaGene™ kit with proteinase K and 1% SDS. Quantity in terms of concentration (ng/µL) was assessed using spectrophotometry and reported using medians and their respective interquartile ranges. (A) DNA obtained by each of the 5 DNA extraction protocols from fresh saliva; (B) DNA obtained by each of the 5 DNA extraction protocols from saliva frozen for 3 months; (C) DNA obtained by each of the 5 DNA extraction protocols from saliva frozen for 6 months; (D) DNA obtained by each of the 5 DNA extraction protocols from saliva frozen for 12 months. Purity was assessed using spectrophotometry and expressed as a percentage of positive samples, absorbance ratio of A260/280 between 1.6 and 2.0 (black fill) or negative, outside the 1.6 to 2.0 range (white fill); (E) Samples from fresh saliva using each of the 5 DNA extraction protocols; (F) Samples from saliva frozen for 3 months using each of the 5 DNA extraction protocols; (G) Samples from saliva frozen for 6 months using each of the 5 DNA extraction protocols; (H) Samples from saliva frozen for 12 months using each of the 5 DNA extraction protocols. Integrity of DNA, visualized as a percentage of unfragmented (black fill), fragmented (white fill) or undetected (gray fill) DNA, as assessed using electrophoretic analysis; (I) Samples from fresh saliva using each of the 5 DNA extraction protocols; (J) Samples from saliva frozen for 3 months using each of the 5 DNA extraction protocols; (K) Samples from saliva frozen for 6 months using each of the 5 DNA extraction protocols; (L) Samples from saliva frozen for 12 months using each of the 5 DNA extraction protocols. Conventional PCR using primers specific for human DNA was used to amplify exon 3 of the interferon regulatory factor 6 (IRF6) gene in all samples at all time points investigated, and then visualized on an agarose gel; samples were either positive (black fill) or negative (white fill) for the presence of amplified human DNA; (M) Human DNA obtained by each of the 5 DNA extraction protocols from fresh saliva; (N) Human DNA obtained by each of the 5 DNA extraction protocols from saliva frozen for 3 months; (O) Human DNA obtained by each of the 5 DNA extraction protocols from saliva frozen for 6 months; (P) Human DNA obtained by each of the 5 DNA extraction protocols from saliva frozen for 12 months. Absolute quantity (µg) was assessed using spectrophotometry and reported using medians and their respective interquartile ranges; (Q) DNA obtained by each of the 5 DNA extraction protocols from fresh saliva; (R) DNA obtained by each of the 5 DNA extraction protocols from saliva frozen for 3 months; (S) DNA obtained by each of the 5 DNA extraction protocols from saliva frozen for 6 months; (T) DNA obtained by each of the 5 DNA extraction protocols from saliva frozen for 12 months. For each test at each time point, all protocols were tested overall (p-value <0.05, Kruskal-Wallis Test) and among one another for significant differences (p-value <0.05, Mann-Whitney U test). Numbers above box plots and columns indicate which protocols were significantly different from each protocol

DNA extraction by each protocol was compared over each time tested and among each protocol using – (1) spectrophotometry to analyze both the quantity yielded and the relative purity compared to RNA and proteins; (2) electrophoresis to analyze the integrity of the DNA in terms of being intact or fragmented; and (3) conventional PCR to analyze whether extracted DNA could be amplified using human specific primers. These results are summarized in Figure 2.

The extraction of genomic DNA from whole saliva using Protocol 1 was stable and fairly efficient at every time point tested. It should be noted that protocol 1 was the only protocol where collections were placed in a suspension buffer. With a final elution volume of 100 µL, the amount of DNA obtained by this protocol ranged between 9.98 µg [16.18] and 6.89 µg [7.16], with no significant differences between any of the tested storage time points (*p*-value=0.776, Kruskal-Wallis test, Figure 2Q to 2T). DNA extracted from fresh saliva (T0) using protocol 1 yielded significantly less DNA, 7.89 µg [5.85], when compared to protocols 4 and 5, yielding approximately 42.50 µg [51.77] (p-value <0.001), and 68.69 µg [51.08] (p-value <0.001), respectively (Figure 2Q). With respect to frozen samples, the DNA yield obtained with protocol 1 was similar to protocols 3 and 5 during time T3 (saliva stored for 3 months), and protocol 4 during T6 (saliva stored for 6 months) and T12 (saliva stored for 12 months), as depicted in Figures 2B to 2D and 2R to 2T.

When compared to protocol 1, protocol 2 consistently extracted less DNA from frozen saliva at all of the investigated time points (*p*-value <0.001, Kruskal-Wallis test, Figures 2B to 2D and 2R to 2T) ranging between 1.09 µg [0.81] and 0.48 µg [0.25]. Moreover, as reported in Figures 2A to 2D and 2Q to 2T, protocol 2 recovered the least amount of DNA when compared to all of the other protocols at all the time point tests with only two exceptions, at fresh saliva and T12. Additionally, at T6 (10 ng/µL [6]) and T12 (10 ng/µL [3]) DNA extractions using protocol 2 were significantly lower when compared to the 35 ng/µL [39] of DNA extracted from fresh saliva using protocol 2 (p-value <0.002, Mann-Whitney U test).

In general, the storage time of saliva affected the concentration of DNA extracted by protocol 3 (Figure 2A to 2D). More specifically, protocol 3 extracted the greatest concentration of DNA from fresh saliva (167 ng/µL [173]) followed by T3 (75 ng/µL [111]), T6 (41 ng/µL [24]) and finally T12 (5 ng/µL [9]). These concentrations of DNA extracted are all significantly different among each other with the exception of T3 versus T6 (p-value=0.074). When DNA was extracted from fresh saliva, the total amount obtained from protocols 1, 2 and 3 were significantly lower than protocols 4 and 5 (p-value <0.0001, Kruskal-Wallis test, Figure 2Q).

The storage time influenced the amount of DNA extracted by protocol 3, the least amount of DNA was extracted using protocol 3 from saliva stored for 12 months, 0.26 µg [0.47], (Figure 2T) when compared to all other times and all other protocols.

Significant differences in the amount of DNA extracted by protocol 4 were found among T0 (250 ng/µL [263]), T3 (172 ng/µL [148]) and at the test times after 3 months (T6; 60 ng/µL [102] and T12; 87 ng/µL [42]) as depicted in Figures 2A to 2D (p-value <0.05, Mann-Whitney U test).

Protocols 4 and 5 were able to extract the greatest amount of DNA from fresh saliva, 42.50 µg [51.77] and 68.69 µg [51.08], respectively, in a final elution volume of approximately 170 µL, when compared to all other protocols and tested time points (p-value <0.0001, Kruskal-Wallis test, Figure 2Q). Additionally, there was a significant difference between the amount of DNA extracted from fresh (T0) versus frozen saliva (T3, T6 and T12) as reported in Figures 2A to 2D (p-value <0.001, Mann-Whitney U test). Lastly, at T12, protocol 5 yielded the greatest amount of DNA 22.67 µg [16.28] in 170 µL when compared to protocol 2 (0.48 µg [0.25] in 50 µL), and 3 (0.26 µg [0.47] in 50 µL) as reported in Figure 2T.

In terms of relative DNA purity compared to RNA and proteins detected by spectrophotometry, no significant differences were detected in the purity of DNA extracted from fresh saliva among protocols 1, 2, 3 and 4 (p-value >0.05, Mann-Whitney U test, Figures 2E to 2H). Conversely, protocol 5 did not have any samples that tested within the 1.6 to 2.0 spectral window from DNA extracted from fresh saliva or saliva that had been frozen for 6 or 12 months (Figure 2E to 2H). DNA extracted from saliva frozen for 3 months had 2 sets of protocols that were significantly different (p-value ≤0.002, Figure 2F); protocols 1 and 2 were not significantly different (p-value =0.686) with about 84% of samples testing within the limits, whereas protocols 3, 4 and 5 were not significantly different (p-value ≥0.327) with less than half the samples testing within the purity limits for each of these protocols (Figure 2F). In general, at all the tested time points, protocols 1 and 2 had the greatest number of samples within the purity limits, while protocols 3 and 4 were variable. The percentage of samples that were found to be within the purity limits was negatively correlated with storage time for protocol 4; conversely the percent of pure DNA samples from the other extraction protocols were not correlated with storage time. Protocol 5 rarely had samples that tested positive for relative DNA purity compared to RNA and proteins, *i.e.*, the ratio of the absorbance at 260 nm and 280 nm was almost always below 1.6.

**Figure 3:**
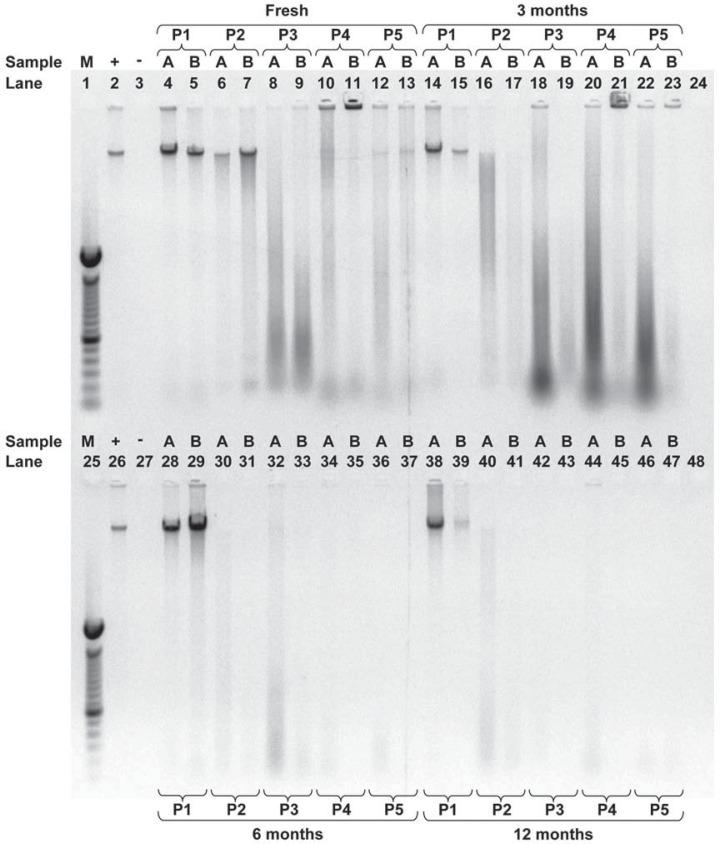
An example of a gel from 5 different extraction protocols when DNA was extracted from fresh saliva or saliva stored for 3, 6 or 12 months investigating whether samples were fragmented. DNA samples from 2 individuals (A, B) were electrophoresed using a 0.8% agarose gel in Tris-acetate (200 mM) with EDTA (50 mM) buffer. Lanes 1 and 25 contain the 100 bp molecular weight standard (M); lanes 2 and 26 contain the positive control (+), 115 ng of human DNA; lanes 3 and 27 contain the negative control (-), 8 µL of ddH2O; lanes 24 and 48 were left blank; all other lanes contain 8 µL of extracted DNA from either volunteer A or B. Protocol 1 (P1) used the Oragene™ kit; protocol 2 (P2) used the QIAamp® DNA Mini kit; protocol 3 (P3) used ammonium acetate, protocol 4 (P4) used the InstaGene™ Matrix kit; protocol 5 (P5) used the InstaGene™ kit with proteinase K and 1% SDS

Electrophoretic analysis with agarose gels was used to further characterize the condition of the extracted DNA. Figure 3 provides a representative example of a gel containing samples from two individuals at every storage time investigated using each of the protocols. When comparing samples taken from fresh DNA, after ≥3 years of storage, DNA extractions from fresh saliva using protocol 1 yielded 100% unfragmented DNA, whereas protocols 2, 3, 4 and 5 yielded 5%, 0%, 10% and 20%, respectively (Figure 2I). It was also found that only protocol 1 yielded 100% or nearly 100% of samples with unfragmented DNA consistently across all the examined time points (Figure 2I to 2L and Figure 3). On the other hand, protocols 2, 3, 4 and 5 rarely had unfragmented DNA and, furthermore, as the storage time of the saliva increased, a greater percentage of DNA samples were undetectable by electrophoresis (Figure 2I to 2L and Figure 3). In particular, the percentage of samples from DNA extractions from saliva stored for one year yielded the following results: protocol 1 had 95% unfragmented DNA and 5% fragmented DNA; protocol 2 had 10% unfragmented DNA, 45% fragmented DNA and 45% undetectable amounts of DNA; protocol 3 had 32% fragmented DNA and 68% undetectable amounts of DNA; protocol 4 had 59% fragmented DNA and 41% undetectable amounts of DNA; and protocol 5 had 38% fragmented DNA and 63% undetectable amounts of DNA (Figure 2L).

Lastly, the extracted DNA was analyzed using conventional PCR and electrophoresis to investigate the percentage of samples that could be amplified by primers that are specific for human DNA. When using fresh saliva, this analysis indicated that 95% of the samples under protocol 1, 100% under protocol 2, 90% under protocol 3, 80% under protocol 4 and 5% under protocol 5 could be amplified with human specific primers (Figure 2M). The storage time of saliva generally did not affect the percentage of samples positive for human DNA in all of the protocols that were tested (Figure 2M to 2P). Only protocols 4 and 5 had a significant difference among the tested time points, both differences were between T6 and T12 (70% versus 90% for protocol 4, respectively; p-value =0.035 and 20% versus 0% for protocol 5, respectively; p-value =0.035). Overall, at nearly all the time points examined, protocols 1 through 4 were not significantly different from one another (p-value >0.05) and these protocols were all significantly greater than protocol 5 (p-value <0.05).

## Discussion

When investigating DNA, the choice of the protocol used to obtain genomic DNA can significantly impact the experiment. A study may require a simplified collection system and the long term storage of samples while maintaining the ability to extract a significant amount of DNA that is relatively pure. Depending on the context, whole saliva has several facets that make it an ideal candidate for extracting DNA. For this purpose, this study investigated the quantity and quality of DNA extracted from whole saliva that was fresh or frozen for 3, 6 or 12 months using 5 different protocols. A complicated picture emerged when taking into account (1) the quantity of DNA extracted, (2) the purity of DNA compared to RNA and protein, (3) the condition of the DNA extracted whether fragmented or unfragmented and, finally, (4) if each protocol extracted significant amounts of human DNA that could be amplified using conventional PCR.

In general, more DNA could be extracted from fresh saliva especially when using protocols 3, 4 and 5. Likewise, fresh saliva generally allowed for greater percentages of samples that were within the standard of relative DNA purity compared to RNA and proteins, as detected by spectrophotometry. Longer storage times generally did not impact the DNA’s integrity, and protocol 1 was much better at extracting DNA that remained unfragmented, whereas protocols 2, 3, 4 and 5 extracted DNA that was almost entirely fragmented or undetectable. In general, the storage time did not influence the percentage of DNA samples amplified by human specific primers in each protocol. Moreover, in every protocol except for protocol 5 most DNA samples were able to be amplified using human specific primers. It remains to be investigated whether procedures in protocol 5 either inhibited conventional PCR or if most of the DNA extracted was nonhuman. Lastly, it should be noted that (1) the final elution volumes can be altered in all of the protocols and that (2) in protocol 2 more DNA can be extracted with more elutions with diminishing concentrations. Therefore, protocol 2 yields a varying amount/concentration of DNA depending on the number of elutions used.

It was expected that protocols 3, 4 and 5 would yield more DNA since they extracted DNA from 1.5 mL of saliva compared to protocols 1 and 2 which used approximately 0.2 mL. It is untested if multiple collections by protocols 1 and 2 using the same amount of starting material, 7.5 times the amount, would truly yield 7.5 times the amount that was collected. However, if this assumption is valid, then protocol 1 would be more efficient at extracting DNA from saliva when compared to protocols 2 and 3 and more comparable to protocols 4 and 5. That is, protocol 1 would have yielded approximately 59.18 µg of DNA from fresh saliva, 74.85 µg of DNA from saliva frozen for 3 months, 51.67 µg of DNA from saliva frozen for 6 months and 70.27 µg of DNA from saliva frozen for 12 months; whereas protocol 2 would have yielded 13.27 µg of DNA from fresh saliva, 8.17 µg of DNA from saliva frozen for 3 months, 3.6 µg of DNA from saliva frozen for 6 months and 3.6 µg of DNA from saliva frozen for 12 months. However, multiple collections from protocols 1 and 2 would further increase the cost and extraction time. It should be stressed that each protocol was being evaluated alone and that all of the facets tested (quantity, purity, integrity and ability to be amplified by human specific primers) should be evaluated when choosing a protocol.

Protocol 1 was the only protocol where saliva was collected and placed into a suspension buffer, and it was also the only protocol where the storage time did not significantly affect the concentration/amount of DNA extracted. Additionally, at every time point tested, the samples of DNA extracted using protocol 1 had the greatest percentages of purity, the most unfragmented samples and nearly all samples tested yielded positive results when using human specific primers. Perhaps the addition of a suspension buffer in protocol 1 was instrumental in preserving the saliva for DNA extraction and keeping the DNA unfragmented regardless of storage time.

Protocol 2 recovered the least amount of DNA when compared to nearly all of the other protocols at all the time points tested. Although, as noted above, additional elutions may yield more overall DNA from the spin column, but this also reduces the concentration. Similarly to protocol 1, the percentage of samples within the purity threshold were unaffected by storage time and most samples were within the accepted threshold for purity. However, only a few DNA samples, at various time points, were unfragmented. As demonstrated by the PCR results in this study, it is still possible to investigate different parameters with fragmented DNA, but caution should be exercised when using protocol 2 for some genetic experiments.

DNA extraction using protocol 3 was sensitive both to saliva being frozen and to being stored for longer periods of time. More specifically, protocol 3 extracted the greatest concentration of DNA from fresh saliva which was significantly greater when compared to saliva that had been frozen for 3, 6 and 12 months. Compared to the other protocols, protocol 3 extracted the least amount of DNA when saliva was stored for 12 months, while DNA extractions from fresh saliva were greater than protocols 1 and 2 (unnormalized for the starting amount of saliva), but less than protocols 4 and 5. The percentage of samples testing within the purity limits was variable among the storage times investigated when using protocol 3. In terms of integrity, similarly to protocols 2, 3 and 5, DNA extracted using protocol 3 was completely fragmented at all of the time points that were investigated with the exception of T6 where 6% of the samples were unfragmented. Lastly, almost all DNA samples extracted using protocol 3 at every time point examined tested positive for human DNA.

Protocol 4 typically yielded the second greatest amount of DNA from saliva samples, and, if strictly looking at human DNA, the most amount of DNA when compared to the other protocols. The purity of DNA extracted using protocol 4 was adversely affected by storage time, and the percentage of DNA samples within the acceptable purity threshold were negatively correlated with storage time. Protocol 4 did extract unfragmented DNA in a few samples, similar to protocols 2, 3 and 5, but not nearly as well as protocol 1. Almost all DNA samples extracted using protocol 4, at every time point examined tested positive for human DNA.

Although protocol 5 yielded the most DNA from fresh saliva, nearly all of the DNA samples extracted using this protocol were not within the acceptable relative DNA limits for purity as assessed by spectrophotometry. Many of the samples must have contained much greater amounts of RNA and/or proteins. Indeed, rarely were DNA samples from protocol 5 found to be unfragmented or even detectable using electrophoretic analysis, and furthermore, most samples were not amplified by PCR using human specific primers. For that matter, it remains unknown precisely how much any protocol may directly interfere with conventional PCR compared to just simply not extracting enough human DNA with sufficient quantity and/or integrity.

As in the present study, there are several other studies that aimed to establish which DNA extraction from saliva protocols were the most efficient^1^ ^,^ ^9^ ^,^ ^12^ ^,^ ^18^ ^,^ ^20^. Commercial kits are generally the most commonly used, generally presenting more consistent results. This consistency of DNA extraction from saliva using the QIAamp DNA kit is apparent when comparing the results of this study with other investigations^18^ ^,^ ^20^. The same can be said when comparing this study’s results to other investigations when looking at the results obtained by using the Oragene Genotek kits^20^. It should be noted that many of these other studies were performed with saliva that was not stored below 0°C.

Briefly, a group of researchers^18^ stored saliva at -20°C for 6 months using the QIAamp DNA kit for DNA extraction. When comparing the concentration and quality of DNA extracted from this saliva to the present study, at the same storage durations, the results are in close agreement. Furthermore, the freezing of saliva for 11 years using the same conditions^6^ with the QIAamp DNA kit also had results similar to those of the present study with saliva frozen for 12 months. Furthermore, the Oragene DNA kit (DNA Genotek) states that viable DNA can be extracted reliably from saliva stored indefinitely at temperatures between -15°C and -20°C and stored for several years at room temperatures. This protocol produced consistent results in terms of quantity and quality at the time points investigated in this study and when compared to other studies in the literature. In particular, the results from this study and a study by Ng, et al.^14^ (2006) had a DNA concentration of about 100 ng/µL with most samples being within the purity threshold based on spectrophotometry.

The commercial kits mentioned above are easy to handle and very consistent, but sometimes unviable due to costs. With this in mind, some studies^1^ ^,^ ^12^, including the present one, tested low-cost alternative protocols such as the protocols that used ammonium acetate or the matrix Instagene Bio-Rad reagent. With respect to the protocol using ammonium acetate, other researchers investigated DNA extraction from saliva stored at room temperature for 1, 2, 4, 8, 15 and 30 days^1^ ^,^ ^12^. These results were similar to what was found in this study regarding both the concentration and quality of DNA. To date, no studies were found that investigated DNA extraction protocols using ammonium acetate where DNA was extracted from saliva that was frozen. Notably, saliva frozen for 6 and 12 months in this study did not efficiently yield DNA when using the ammonium acetate protocol, and thus, studies hoping to extract intact DNA in sufficient quantities from frozen saliva should not use this protocol. The other low-cost protocol using the Instagene Matrix from Bio-Rad had intriguing results, extracting large amounts of DNA but with less quality/purity when compared to some of the other protocols used. However, as time storage of the saliva increased both the quantity and quality of the DNA extracted diminished.

A study by Goode, et al.^8^ (2014) details an optimized procedure for extracting DNA using reagents from the Puregene extraction kit (Qiagen)^8^. They found that using a reagent volume smaller then recommend by the manufacturer did not compromise the amount of DNA extracted and optimized costs. Notably, their protocol is similar to protocol 3 used in this study. Further examination may reveal that costs and DNA extractions can be further optimized in a similar manner.

A search of the literature revealed only one DNA extraction from microorganisms from cultured mediums using the Instagene matrix protocol^3^ ^,^ ^10^. This study noted the successful extraction of DNA from this source, but it remains difficult to compare this result with the present study where the extractions were from whole saliva.

## Conclusion

When viewed with the perspective gained from this study and from other independent studies, it is estimated that commercial kits, sometimes independent of the storage time, provide consistent results in terms of the concentration and purity of DNA extracted from whole saliva, especially when dealing with saliva that has been frozen and/or stored for a long time. If whole genomic DNA is needed, then only protocol 1 can be recommended. The less expensive laboratory protocols provided less sufficient results with stored frozen saliva; however, fresh saliva or saliva stored for short durations might be adequate for obtaining DNA with a sufficient quantity and purity albeit fragmented. Although, as stressed above, a complicated picture emerges when taking into account the extracted DNA’s quantity, purity, quality and the protocols ability to provide decent starting material for PCR, and, depending on a given researchers needs, one protocol’s particular strengths and costs might be the deciding factor for its employment.
